# Major air pollutants seasonal variation analysis and long-range transport of PM_10_ in an urban environment with specific climate condition in Transylvania (Romania)

**DOI:** 10.1007/s11356-020-09838-2

**Published:** 2020-07-03

**Authors:** Zsolt Bodor, Katalin Bodor, Ágnes Keresztesi, Róbert Szép

**Affiliations:** 1 Department of Bioengineering, Sapientia Hungarian University of Transylvania, Piaţa Libertăţii 1, 530104 Miercurea Ciuc, Romania; 2 Institute for Research and Development for Hunting and Mountain Resources, str. Progresului, 35/B, 530240 Miercurea Ciuc, Romania; 3 Faculty of Natural Sciences, Doctoral School of Chemistry, University of Pécs, Ifjúság 6, Pécs, 7624 Hungary

**Keywords:** Seasonal variation, Backward trajectory, Cluster analysis, PM_10_, Air pollution, CWT

## Abstract

The air quality decrease, especially in urban areas, is related to local-scale conditions and to dispersion of air pollutants (regional and long-range) as well. The main objective of this study was to decipher the seasonal variation of PM_10_, NO, NO_2_, NO_*x*_, SO_2_, O_3_, and CO over a 1-year period (2017) and the possible relationships between air pollution and meteorological variables. Furthermore, trajectory cluster analysis and concentration-weighted trajectory (CWT) methods were used to assess the trajectories and the source-receptor relationship of PM_10_ in the Ciuc basin Transylvania, known as the “Cold Pole” of Romania. The pollutants show lower concentrations during warmer periods, especially during summer, and significantly higher concentrations were observed on heating season in winter due to seasonal variations in energy use (biomass burning) and atmospheric stability. Subsequently, in February, the highest concentration of PM_10_ was 132 μg/m^3^, which is 4 times higher than the highest recorded monthly mean. Our results indicate a negative correlation between CO/temperature (− 0.89), NO_*x*_/temperature (− 0.84) and positive between NO_*x*_/PM_10_ (0.95), CO/PM_10_ (0.9), and NO_*x*_/CO (0.98), respectively. Dominant transport pathways were identified and the results revealed that slow-moving southerly (~ 45%) and northwesterly (~ 32%) air masses represent almost 80% and mainly regional flows were discerned. During 2017, increased PM_10_ levels were measured at the study site when air masses arrived mostly from northwest and southeast. The CWT and polarplot models show a strong seasonal variation and significant differences were observed between weekdays and weekends, namely highest PM_10_ concentrations during weekends at low wind speed (2–4 m/s).

**Electronic supplementary material:**

The online version of this article (10.1007/s11356-020-09838-2) contains supplementary material, which is available to authorized users.

## Introduction

In the twenty-first century, one of the biggest challenges is air pollution, not only at global scale but also at local and regional levels. Air pollution may vary from minutes to decades (Liu et al. 2011, 2013; Wang et al. 2017; Reizer and Orza 2018), therefore it is a major health, social and economic problem (Jeong et al. 2011; Russo et al. 2014; De Marco et al. 2019; Hong et al. 2019). The major concern of humankind about air quality is to decipher the connection between anthropogenic activities and air pollution as long as by the year 2050 it will become the world major environmental cause of mortality (OECD 2012 2014). On the other hand, the problems related to particulate matter (PM_10_ and PM_2.5_ μm) (Makra et al. 2011; Bodor et al. 2020), resulting from urbanization as well as from industrialization, have steadily created a real need for a well-elaborated and efficient long-term emission controls (Al-jeelani 2016). Hence, in order to control and maintain air quality, it is mandatory to determine the potential pollution sources and emissions both locally and globally. For example, the PM_10_ mean value for short-term (24-h mean) is 50 μg/m^3^ (WHO 2006, 2016) and in the urban environment is frequently exceeded especially if there is a strong industrial background nearby, increased traffic activities, or strong and persisted inversions during winter (Chen et al. 2015a; Cichowicz et al. 2017). Therefore, to understand even at a regional level the air pollutants origin, seasonal variation, and characteristics is an important issue (Park et al. 2015). Previous studies have revealed that air pollution not only affects climate (Jeong et al. 2011; Cholakian et al. 2019) but subsequently many health problems are linked to high PM concentrations due to short- and long-term exposure (Brunekreef and Holgate 2002; Makra et al. 2011; Yang et al. 2015; Cha et al. 2017; Faridi et al. 2018; Bennett et al. 2019; Zhong et al. 2019). Regarding the origin of pollutants, they can be of either anthropogenic or natural, for example, desert dust has a major contribution to PM concentration increase all over in Europe (Ryall et al. 2002; Valenzuela et al. 2012). Several authors have investigated the origin of PM_10_ in different European regions (Querol et al. 2004; Makra et al. 2013; Russo et al. 2014) by collecting data on air pollutant release. Furthermore, Hellack and colleagues (Hellack et al. 2015) have proved the impact of long-range transport on urban air quality. According to the results, approximately 50% can be mainly attributed to the air parcel movement. Possible long-range sources with an impact on the collected rain samples were presented by Uygur et al. (2010) and Keresztesi et al. (2019, 2020a, b). Thus, to determine long-range transport of major pollutants, potential sources and the relationship between pollutants and meteorological parameters (Demuzere et al. 2009) are of great importance. In the last few decades, a huge amount of air quality data at different levels have been stored and currently most of the data are freely available and can be used to determine and model air pollution and potential emission sources (Munir 2016; Czernecki et al. 2017; Szulecka et al. 2017). Consequently, modeling approaches are widely used in many areas to evaluate the origins, trajectories, and the dispersion of pollutants.

Transport pathways of air masses and the potential source areas can be identified using back-trajectory analysis (e.g., clustering trajectories) (Draxler and Rolph 2014; Stein et al. 2015) and concentration-weighted trajectory (CWT) (Carslaw 2015, 2018) simulations. For example, back-trajectory analysis approaches were applied in different studies to analyze the long-range transport impact on air pollution (Jeong et al. 2011; Makra et al. 2013; Tahri et al. 2017). Moreover, potential source contribution function (PSCF) and CWT methods can be successfully implemented to investigate the origins of pollutants (source areas) (Cheng et al. 2013; Hao et al. 2019). Briefly, back trajectories and long-term measurements are combined (Eq. 1) and source areas with potential impact on air quality are deciphered by plotting the results on a map.

Regarding the air pollution in our region, Szép and colleagues (Szép and Mátyás 2014; Szép et al. 2016, 2017b, 2019) investigated the PM pollution in the Ciuc basin; however, there are little or no studies on the transport pathways and potential source regions that can potentially affect air quality in this region. Thus, the seasonality aspects of major air pollutants, the correlations between pollutants and environmental parameters, the impact of long-range transport, and the pollution source areas are yet to be discovered.

To address these issues, we collected on an hourly basis the data regarding air pollution from January to December 2017 in the Ciuc basin to analyze the air quality and the most important air pollutant temporal variation, correlations, possible transport patterns, and potential source regions with negative impact on PM_10_ concentration in intra-mountain basins. Finally, the extreme episodes, days with concentrations exceeding the daily limit value were also identified and analyzed.

## Materials and methods

### Site description (Ciuc basin—Miercurea Ciuc, Romania)

**Fig. 1:**
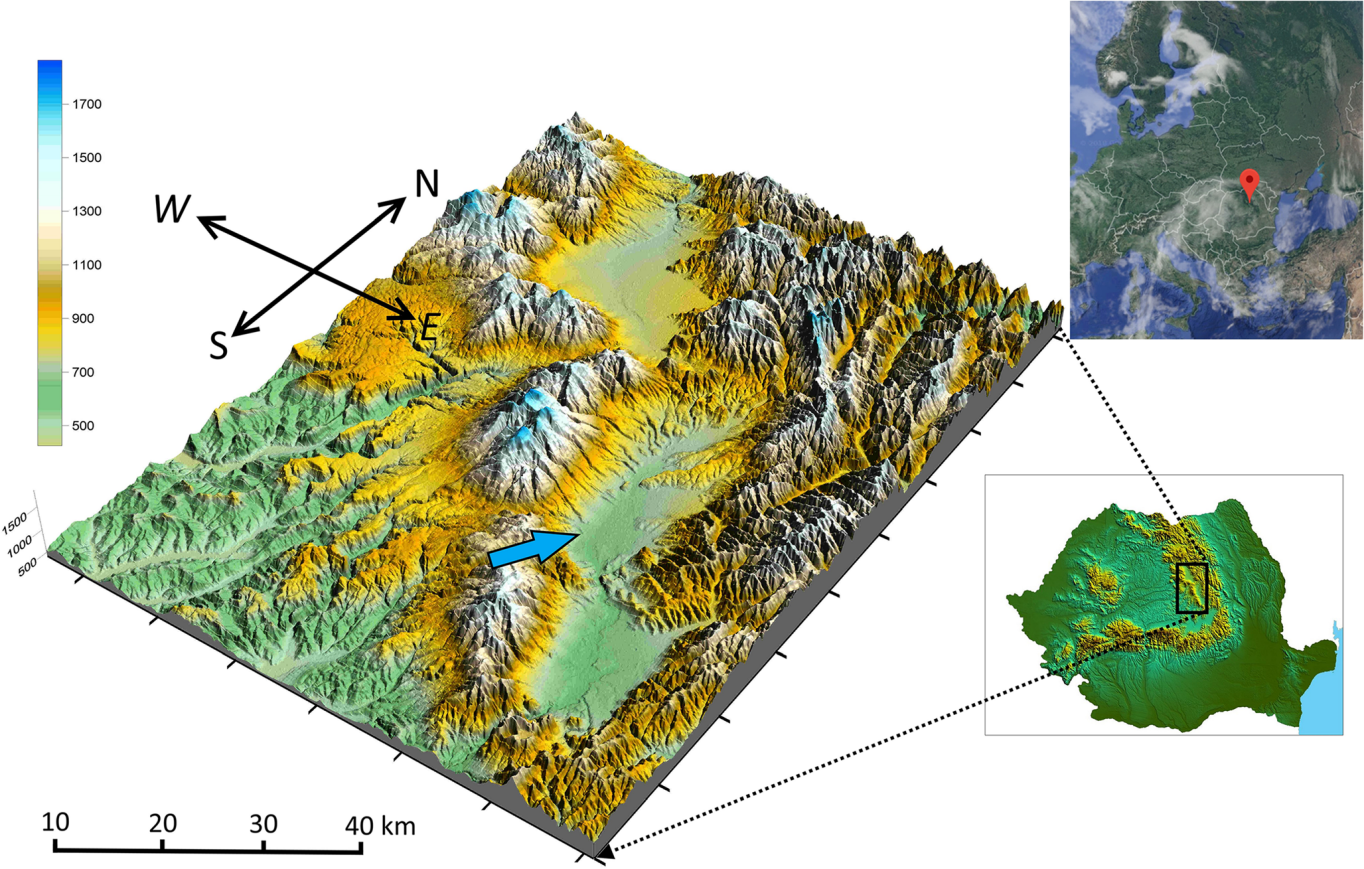
Geolocation of the sampling site (https://www.google.com/maps/). Reproduced and modified from (Szép et al. 2019) with permission

Miercurea Ciuc (46′ 22″ N, 25′ 44″ E, ~ 600 m asl) (Fig. 1) is a small city in Romania, the largest in Harghita County with a population of ~ 40,000. The city is situated in eastern Transylvania (Eastern Carpathians), enclosed by mountains, Harghita Mountains (1800 m) to the west and Ciucului Mountains (1300–1400 m) to the east (Szép et al. 2017b). The study site (Ciuc basin) is situated in the center region of the East Carpathians characterized by a special microclimate as a result of geographical and climate conditions, with frequent and long episodes of static stability, involving strong nocturnal and winter thermal inversions (especially during the cold period), which favors the accumulation of pollutants. Summers are chilly with abundant precipitations while winters are cold which makes this area the coldest in Romania (also called as the Carpathians cold pole). The average annual temperature is only 6.51°C, the minimum relative humidity is 43%, and the reported dominant wind direction is westerly and northwesterly (Kristó 1994). Typically, there are four distinct seasons with long and cold winters and relatively short summers. There are no large industries inside the basin; however, there is an active solid waste landfill situated ~ 30 km away from the station in a high plateau (874 m asl). The monitoring station is located in the southern part of the city.

Due to the EU regulations (Directive 2008/50/EC), Romania is covered by air monitoring stations of all types; hence, the National Air Quality Monitoring Network as a complex system is used to monitor air quality.

Raw data was collected between January 2017 and December 2017 using the urban background station and during measurements the concentration of different pollutants (PM_10_, O_3_, SO_2_, NO_2_, CO, NO_x_ and NO) was followed on hourly basis in the Ciuc basin. Measurements were carried out using the following reference methods: gravimetric method for PM_10_ concentrations (Standard EN 12341); SO_2_ by ultraviolet fluorescence (SR EN 14212); NO, NO_2_, and NO_*x*_ by chemiluminescence (SR EN 14211); O_3_ by ultraviolet photometry (SR EN 14625); and CO by non-dispersive infrared spectroscopy (SR EN 14626), respectively. Regarding the meteorological parameters, wind speed (WS), atmospheric pressure (P), air temperature (T), relative humidity (RH), and rainfall data were collected as well.

One of the major questions about air pollution is the level of local and regional contribution to PM_10_ concentrations. To investigate this issue, the datasets were analyzed statistically and the possible relationship between PM_10_ and other gaseous species (O_3_, SO_2_, NO_2_, CO, NO_*x*_, and NO) was determined using the Spearman’s correlation coefficient. Monthly boxplots were used for each component to decipher the median, mean, first, and third quartile during the study period. The temporal variation of pollutants was paired with different meteorological parameters to elucidate the nature of relationships.

### Back-trajectory analysis

Mathematical models are widely used in many sciences, e.g., to analyze the processes taking place in the atmosphere, to make predictions, or to study source-receptor relationships (Labzovskii et al. 2014; Li et al. 2017; Szép et al. 2019). One of the most extensively used air quality models is the Hybrid Single-Particle Lagrangian Integrated Trajectory (HYSPLIT, http://www.arl.noaa.gov/HYSPLIT_info.php) (Draxler and Hess 1997; Draxler and Rolph 2013; Stein et al. 2015). By implementing this model, the possible paths of air masses and trajectories, as well as complex dispersions and depositions, can be estimated; hence, it can calculate whether the air parcel trajectory is present or not. During simulations, the PC version of the HYSPLIT model (Stein et al. 2015) with an external meteorological data was used. The output from the Global Data Assimilation System (GDAS) has 1° horizontal resolution (approximately 110 × 110 km horizontal and 3-h temporal resolution) (Su et al. 2015). The particle position was obtained from the 3D velocity vectors and linear interpolation was carried out for both space and time.

In our study, 72-h back trajectories were calculated arriving at the study site at different heights, 10, 500, and 1000 m agl at 1200 UTC for specific PM_10_ episodes, January 29 and February 02 2017, exceeding the daily limit value. The idea behind arrival heights selected to be analyzed was that, according to the literature (Hsu et al. 2003), these arrival heights have the largest influence on the PM and other air pollutant concentration. The transport patterns were identified by taking into consideration the trajectories obtained during simulations.

### Statistical and cluster analysis of back trajectories

A single backward trajectory has limited significance (Stohl 1998); therefore, to overcome the limitations and errors, cluster analysis was applied, a multivariate statistical analysis technique (Sokolov et al. 2016; Xin et al. 2016; Tahri et al. 2017) to decipher the most important transport patterns (clusters). A new backward trajectory (72 h) was started every 6 h resulting in a total of 1460 trajectories, using the stand-alone version of the HYSPLIT. In order to evaluate the seasonality aspect trajectories were grouped into four seasons: spring (March, April, May—MAM), summer (June, July, August—JJA), autumn (September, October, November—SON), and winter (January, February, December—JFD), respectively. Simulations were carried out for air parcels arriving at 10 m agl at 1200 UTC; hence, cluster analyses are presented and discussed only for this altitude.

During clustering analysis the trajectories with similar characteristics, based on the geometric distance or total spatial variance (TSV) (Draxler and Rolph 2014), were grouped into different clusters. As a result, the dominant air pathways, with the highest frequency, were identified.

To identify the possible source areas, potentially affecting regional PM_10_ concentrations in the Ciuc basin, HYSPLIT trajectories, PSCF, and CWT were implemented by combining the trajectories with the daily PM_10_ concentration data (Liu et al. 2011). As stated out earlier (Hsu et al. 2003; Xin et al. 2016; Li et al. 2017), one of the main disadvantages of PSCF is that it is difficult to distinguish if the pollution source has moderate or strong characteristics. Therefore, to overcome these problems, the CWT was applied to decipher the possible origin of pollutants (Hsu et al. 2003). Briefly, in each grid cell, there is a trajectory and a mean weighted concentration association (Carslaw 2015). Similar to PSCF, in CWT an arbitrary weight function is employed to minimize or eliminate grid cells with few endpoints. Hence, the main difference between PSCF and CWT is that in this case, every concentration is connected to a back trajectory. Moreover, CWT is more robust to low pollutant concentrations and it measures the level of pollution of a grid cell according to Eq. 1 (Jeong et al. 2011; Kabashnikov et al. 2011; Cheng et al. 2013):

1 $$ {\mathrm{CWT}}_{i,j}=\frac{\sum_{l=1}^L{C}_l{\tau}_{i,j,l}}{\sum_{l=1}^L{\tau}_{i,j,l}} $$

CWT_*i,j*_: CWT value of grid *i*, *j* (latitude, longitude); *C*_*l*_ is the PM_10_ concentration corresponding to the arrival of back trajectory *l*; *L* representing the total number of back trajectories; *τ*_*i,j,l*_ is the endpoint number of back trajectory *l* in grid *i*, *j* (residence time of a trajectory in each grid cell). CWTs for PM_10_ were determined on seasonal basis (spring, summer, autumn, winter) as well as for 2017. The identification of seasonal aspects is essential as long as the Ciuc basin is characterized by strong atmospheric stability (especially during winter), which may affect the CWTs as well. Furthermore, the weekday/weekend effect on PM_10_ was assessed using the bivariate polarplot diagrams of the “openair” R package (Carslaw and Ropkins 2012; Ropkins and Carslaw 2012; Carslaw 2015).

## Results and discussions

**Fig. 2:**
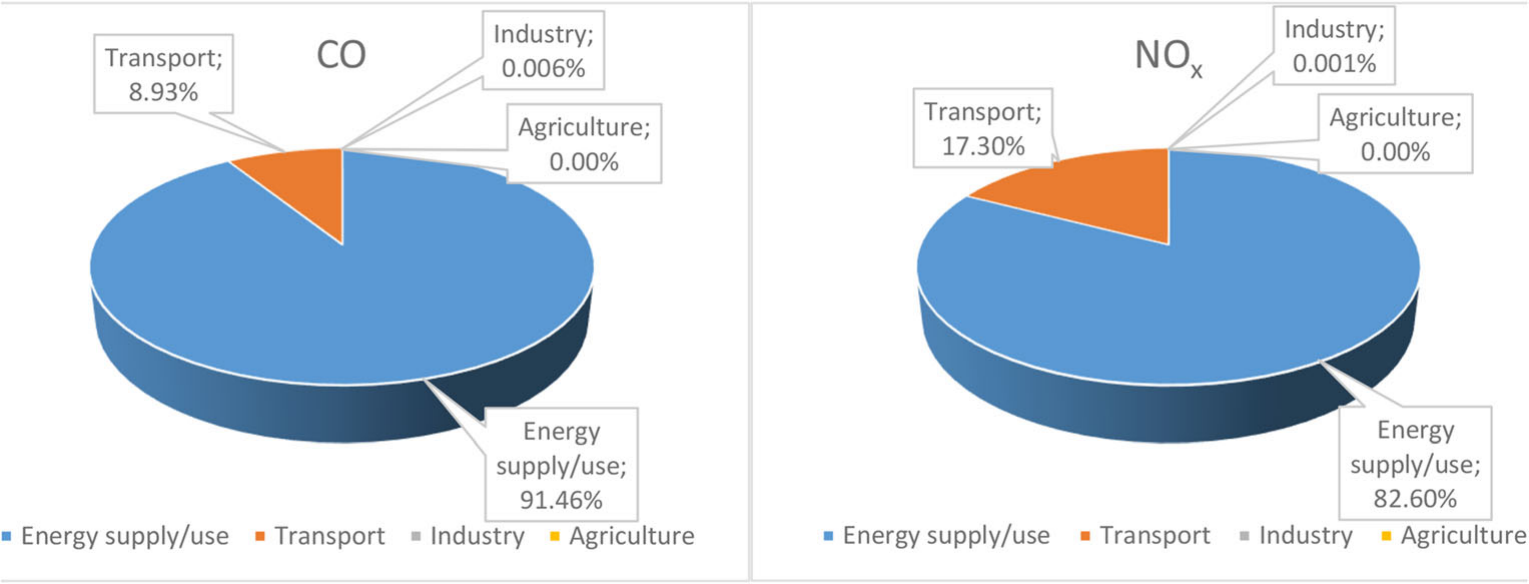
The relative contribution of the emission sources to CO and NO_*x*_ concentrations in 2017

The relationship between meteorological parameters and major atmospheric pollutants was assessed by using monthly average values. Monthly variations of the selected pollutants during the year 2017 in the Ciuc basin are presented in Fig. 2. According to the results, a clear seasonal variation was observed. Furthermore, the results are fairly similar to those reported by Chen et al. (2015b) and Al-jeelani (2016)), namely the concentration of particulate matter increases from October to February in accordance with heating time and strong thermal inversion; therefore, the highest monthly average values were recorded in December (17.04 μg/m^3^), January (28.30 μg/m^3^), and February (34.31 μg/m^3^), respectively. Given these values, the PM_10_ concentrations were statistically higher in cold season than those in warmer season (*p* < 0.05). On the other hand, these results confirmed the strong seasonality aspects of PM_10_, highest levels in winter and lowest levels in spring, summer, and fall. There is a tendency of growth for each pollutant (except O_3_) starting from October to February, then a significant decrease can be observed in the concentrations. The monthly variation of O_3_ concentrations was inversely related to that of PM_10_; hence, in early spring and summer (from April to July), the highest O_3_ levels were recorded, while the minimum concentrations were observed during late autumn and winter, especially in November. Regarding the gaseous pollutants, the trend in case of SO_2_ is similar to that of PM_10_, namely increased levels during cold season (February 5.83 μg/m^3^) and lowest values without significant differences during the rest of the year (July 4.37 μg/m^3^). The NO species (NO, NO_2_, and NO_*x*_) monthly variation pattern follow a similar trend like PM_10_ or SO_2_. Regarding the concentration, tendencies in the case of CO similar variations were reported in the literature (Park et al. 2015). Concentration changes are much higher for CO between winter and the rest of the months and increases sharply from October to January, in analogous to other air pollutants. It is obvious that air quality decreases during winter months; hence, the worst air qualities are recorded during this period; meanwhile, months with low pollution levels are recorded during warmer periods, especially during summer months. Taking into account the results presented in the literature, usually the main emission sources of air pollutants are as follows: NO_2_—from power plants and automobiles; CO—from vehicles, peat, and biomass burning; SO_2_—are from non-renewable fuel burning; and PM_10_—from natural and anthropogenic activities.

Furthermore, it is well documented that the primary sources of atmospheric pollutants can be classified into two main categories: natural and anthropogenic sources; moreover, anthropogenic emissions are mostly related to sustain the population energy and food need. It should be also mentioned that human-made emissions are very diverse and the air pollutant emissions and dispersion vary greatly with time, mainly influenced by the environmental conditions. There are many different types of sources, but the most important emissions come from energy supply/use (production and distribution; the use of energy in industrial, commercial, institutional, and household sectors; non-road transport—mobile equipment used in agriculture and forestry), transport, industry, and agricultural activities, respectively. According to Harghita’s Environmental Protection Agency (APM), significant differences were identified in the relative contribution of the major sectors to CO and NO_*x*_ concentrations in the study area during 2017. The contribution of each sector is presented in Fig. 2.

The results revealed that the main anthropogenic sources of CO and NO_*x*_ are the energy and transport sector with a relative apportionment of ~ 90–83% and 9–17%, respectively. On the other hand, the contribution of industrial activities is very low or even undetectable in the case of agricultural activities, which can be understood in the light of the current situation characterizing this region, no large industrial facilities are located near this area; moreover, agriculture is the largest source of NH_3_.

**Fig. 3:**
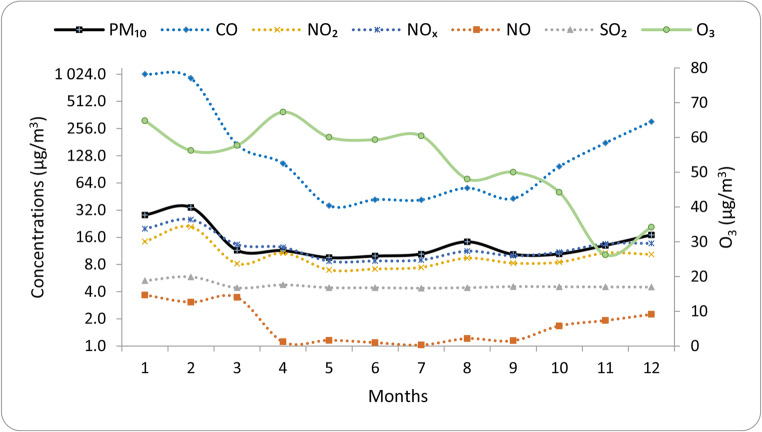
Monthly variation of CO, SO_2_, NO, NO_2_, NO_*x*_, O_3_, and PM_10_ concentrations during 1-year period

The emission sources and relative contribution of each sector to PM_10_ concentration in 2017 were as follows: energy aprox. 90%, traffic 1.6%, industry 0.012%, agriculture 0.063%, and other sectors 7.4%. Almost 90% of this emission occurs in energy-related sectors and within this sector, four areas with contribution to the emissions of suspended particles can be identified: domestic and institutional heating (80% and 1.4%, respectively), traffic (9.6%), and industrial emissions (0.004%). Traffic is a source category that includes different kinds of emissions from various vehicle types; hence, five different categories were identified, namely small cars, vans, heavy vehicles, motorcycles, and railways. The total PM_10_ emissions from road transport were distributed by categories as follows: heavy vehicles, including busses, 43.5%, small cars 31.5%, vans 19.9%, motorcycles 0.55%, and railway transport 4.62%, respectively. The monthly concentration variation of the major air pollutants is depicted in Fig. 3.

The negative correlation of O_3_ with air pollutants can be understood in the light of titration processes taking place between NO and O_3_, and as a result, NO_2_ is formed during this process which is a major contributor to the formation of O_3_ and O in the atmosphere (Latif et al. 2012). Furthermore, high-ozone concentrations are regulated by the association of key climatic factors, including a stable boundary layer, clear skies, high temperatures, and winds (Saavedra et al. 2012), respectively. For example, due to photochemical reactions, surface ozone level will increase; therefore, the positive correlation with wind speed can be explained by the inflow of precursors.

**Fig. 4:**
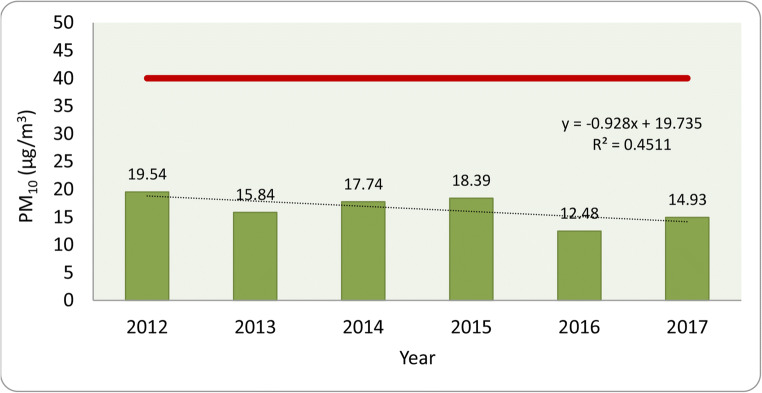
Evolution of PM_10_ concentrations over the last 6 years. The red line represents the annual limit value (40 μg/m^3^)

Regarding the evolution of major air pollutant concentrations for the last 6 years, small changes were detected, for example, in the case of PM_10_, there is a decreasing tendency (Fig. 4). According to the annual mean values, there is a clear trend, while in 2012, the mean PM_10_ concentration was 19.54 μg/m^3^; in 2017, it was only 14.93 μg/m^3^ which is almost a 24% decrease.

**Fig. 5:**
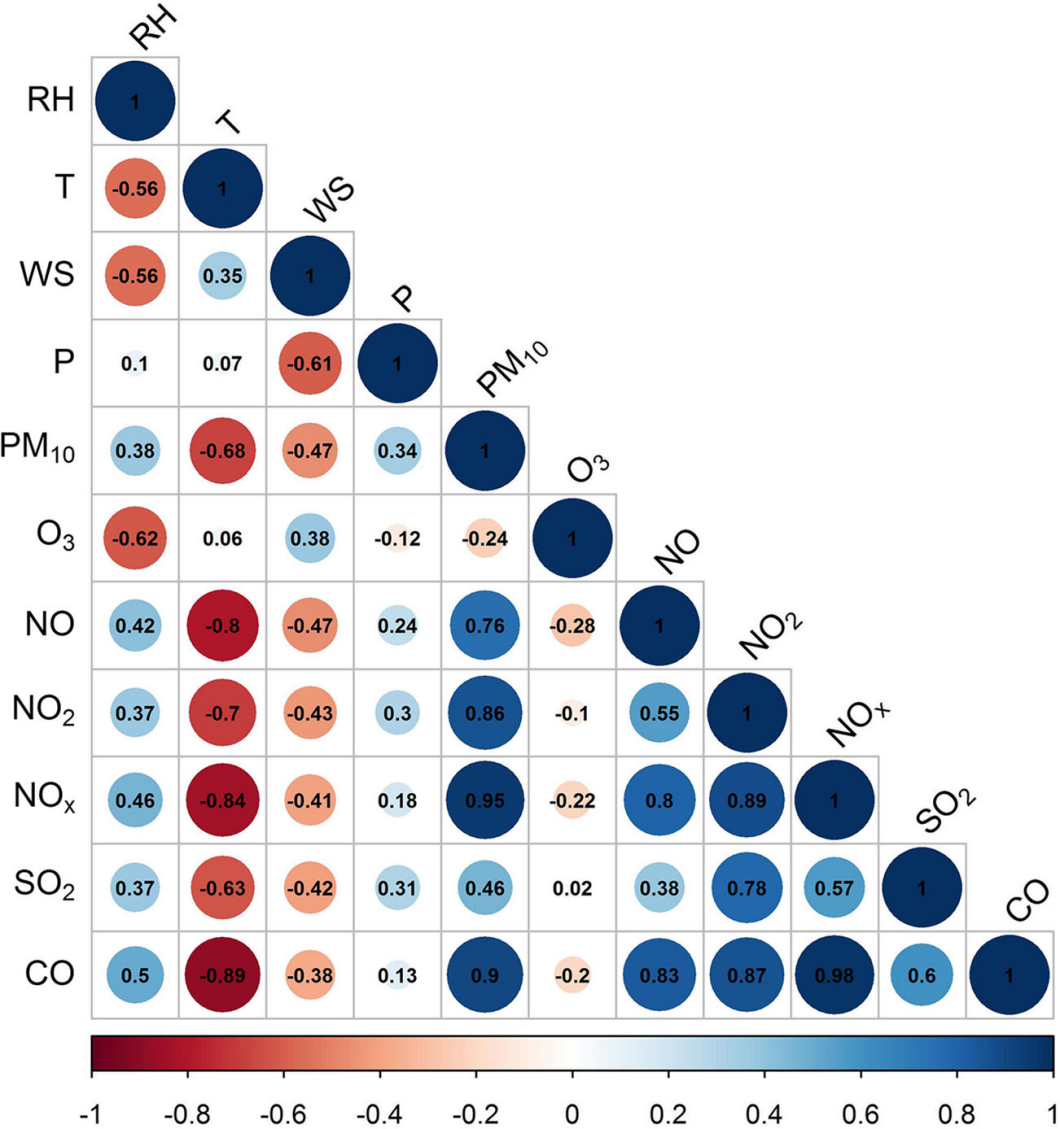
Spearman correlation coefficients obtained for 1-year period

Spearman’s correlation rank analysis was carried out to decipher and quantify the possible relationships between different pollutants and meteorological parameters. Figure 5. shows the correlation coefficients for the study period. The parameters min, max, average, standard deviation, and median were determined for all pollutants; the results are presented in Online Resource 1.

The Spearman rank correlation results revealed a relatively strong positive correlation between PM_10_ and other pollutants such as CO, NO, NO_2_, and NO_*x*_, and moderate between PM_10_ and SO_2_, respectively. On the other hand, moderate negative correlations were observed between O_3_ and other air pollutants; the only difference seems to be in the case of SO_2_ where no correlation was found. Furthermore, a strong negative correlation was between pollutants and environmental parameters such as temperature and wind speed; meanwhile, air pollutants showed a positive correlation with relative humidity and pressure. The monthly Spearman correlation analysis between PM_10_ and air pollutants is presented in Online Resource 2.

### Relationship between meteorological factors and major urban pollutants

**Fig. 6:**
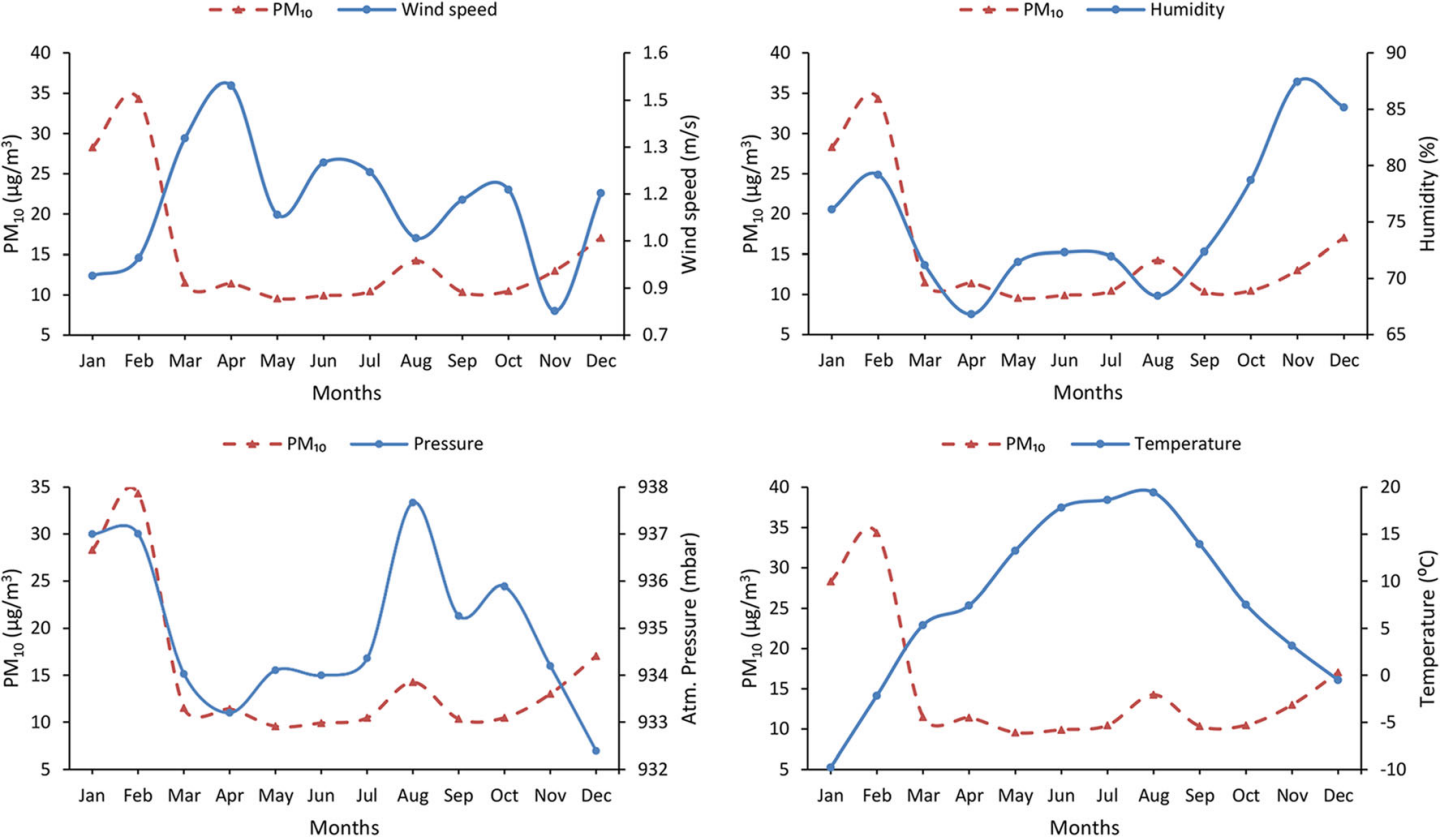
PM_10_, wind speed, humidity, atmospheric pressure, and temperature monthly variations

The key factors that usually have an important impact on PM_10_ concentrations are meteorological parameters, including atmospheric pressure, temperature, wind speed, and humidity. Monthly average values for major air pollutants and meteorological factors were determined during the study period in the Ciuc basin and Fig. 6 depicts the trends and relationships between PM_10_ and key meteorological variables. The results for the other pollutants are presented in Online Resource 3.

A clear seasonal pattern was observed for PM_10_, wind speed, humidity, temperature, and rainfall, respectively. The discerned valleys and peaks indicate the relationship (positive, negative) between parameters. As shown in Fig. 6, a negative association was identified between PM_10_/wind speed (*R* = − 0.469) and PM_10_/temperature (*R* = − 0.678); meanwhile, positive relation (*R* = 0.343) was identified between PM_10_ and atmospheric pressure. The main reason is that during high pressure, an anticyclone-like system will form; therefore, the wind speed decreases and a stable atmospheric condition is reached; thus, the dispersion level is also significantly reduced. In the case of humidity, a seasonal correlation was observed, positive relation during cold periods and negative during warm periods. Regarding the temperature correlation, one reason could be the effect of solar heating, as long as during daytime the dispersion of aerosols is faster. Moreover, the impact of traffic on air pollution, which is a major contributor to local, regional, and global air pollution (Colvile et al. 2001; Salvi and Salim 2019) is well known. For example, Park and colleagues (Park et al. 2015) found that CO concentration is greatly affected by human activities, especially by the number of cars. As long as the number of registered cars will increase in the future, it is expected to increase the CO concentration as well.

**Fig. 7:**
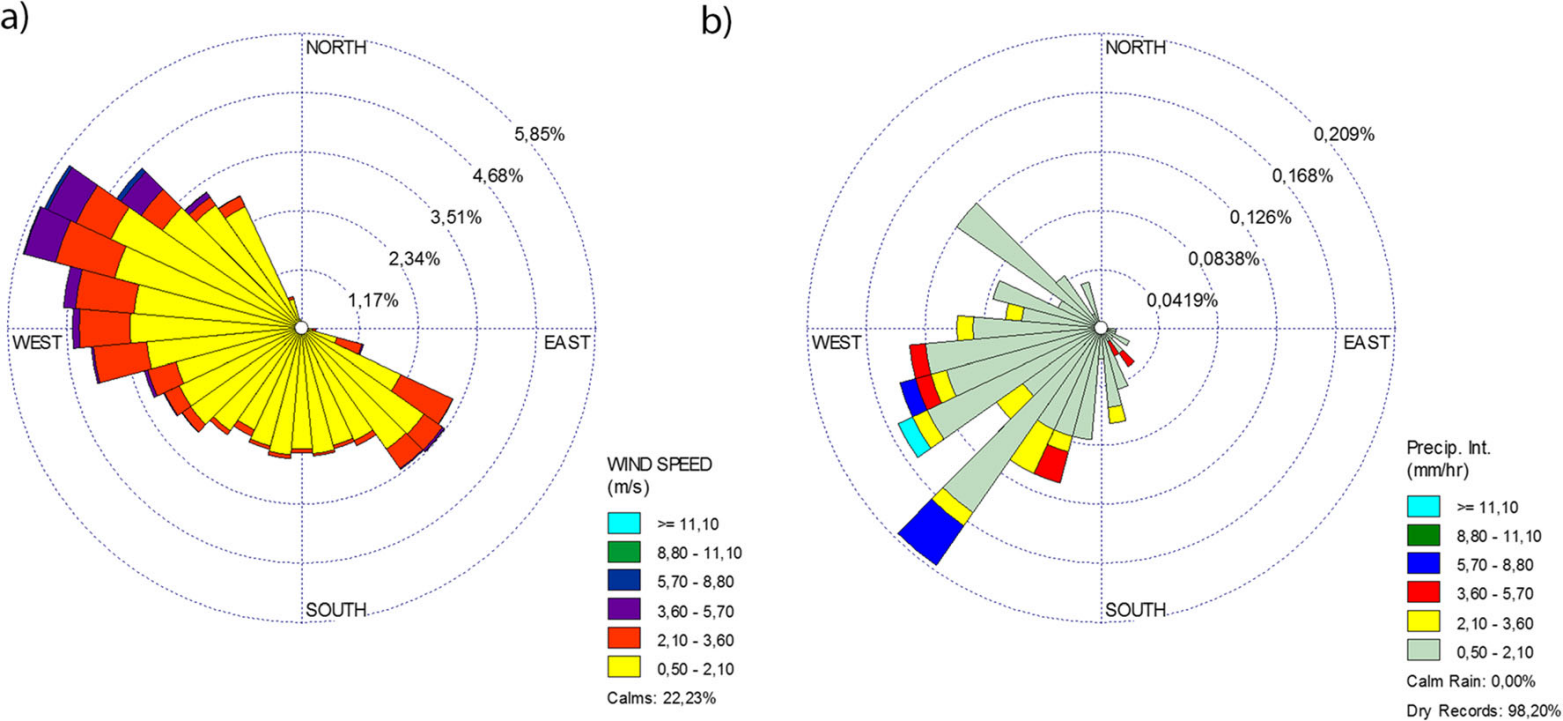
Wind (**a**) and rain rose (**b**) depicting frequency distribution of wind and precipitation for the entire period

The dominant wind and precipitation directions, as well as intensities, were identified using the well-known wind rose and rain rose diagrams (Fig. 7).

The results revealed that the average wind speed was 1.07 m/s in northwest, southwest, and southeast directions, respectively. On the other hand, the higher wind speeds were associated with northwest directions. Regarding the precipitation directions and intensities, the results show that the highest occurrences of rains were attributed mainly to winds with low speed and from southwest directions.

**Fig. 8:**
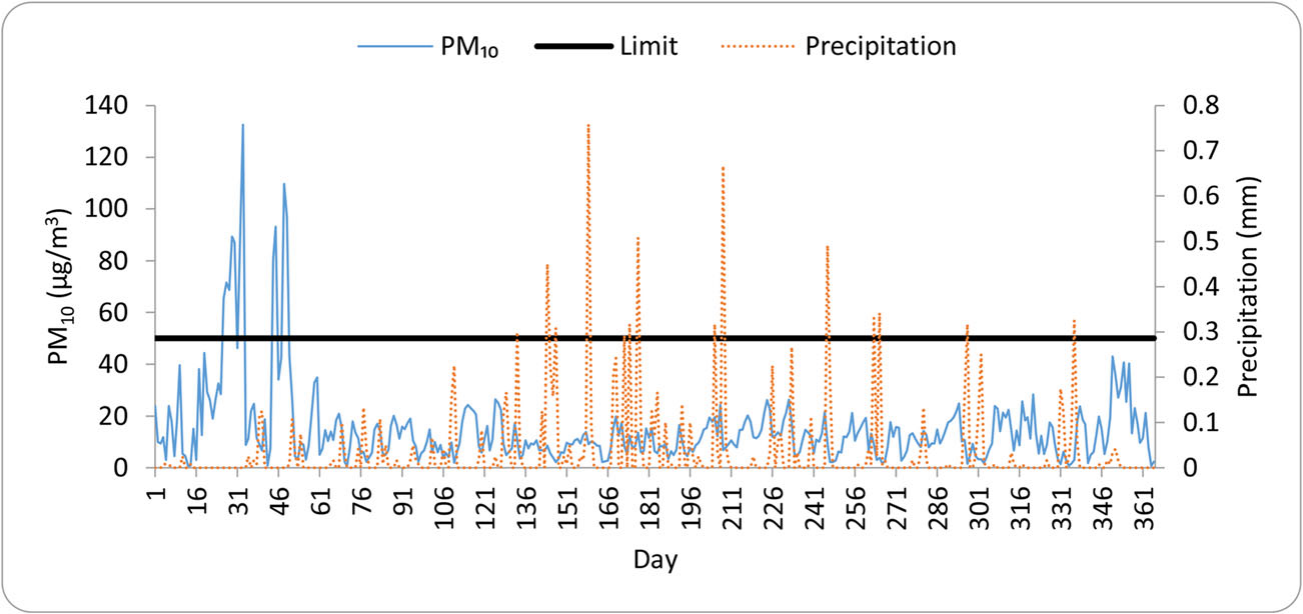
Daily variability of PM_10_ and precipitation during the study period. Black line represents the daily limit value (50 μg/m^3^)

Since precipitation plays a key role in pollutant removal, due to the washout effect, a negative correlation of − 0.839 was observed between PM_10_ and precipitation during the study period. This is one of the main reasons why the pollution level is usually lowest in spring and summer when the highest daily amount of precipitation and the highest rainfall frequency were recorded. Figure 8 depicts the daily time series data for PM_10_ and precipitation in the study region during the whole sampling period (2017), as well as the daily limit value of PM_10_. According to the results, it was discerned that during the sampling period, 11 events exceeded the legal daily limit value (50 μg/m^3^). The highest daily mean of PM_10_ recorded in this region was 132.58 μg/m^3^ on February 02 and the lowest was 0.64 μg/m^3^ on March 10. Nonetheless, the 1-year average loading was far below the admissible limit (40 μg/m^3^).

Time series plots of the measured parameters confirm that the exceeding periods were in January and February. As presented in Fig. 8, the concentration of PM_10_ is mostly influenced by precipitation and precipitation quantities, known as wet deposition; hence, a significant negative correlation can be identified with all pollutants. Therefore, as was expected, the highest concentrations were measured during non-rainy days (Querol et al. 2009).

### Seasonal variation of atmospheric pollution

**Fig. 9:**
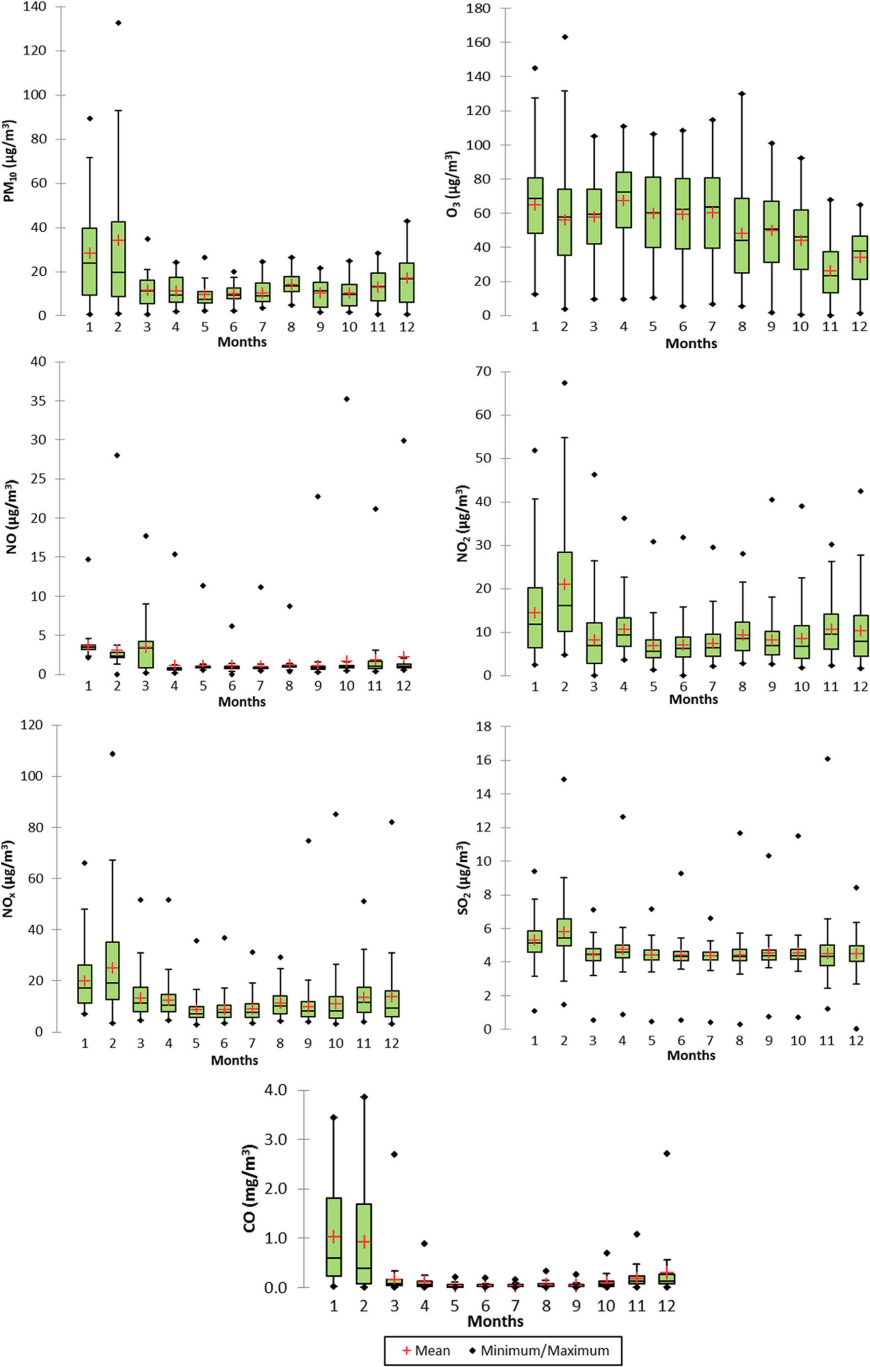
Boxplots of PM_10_, O_3_, NO, NO_2_, NO_*x*_, SO_2_ (μg/m^3^), and CO (mg/m^3^) concentrations for each month during the year 2017. The means are represented by red crosses and the medians by black horizontal bars. Box lower and upper limits represent the first and third quartiles. The ends of the whiskers represent the one SD above and below the mean, and any value not included between the whiskers is represented as an outlier with a black point

The seasonal variation and evolution of the monthly values of each species were evaluated by using descriptive statistics. The results obtained are presented as boxplots in Fig. 9, where monthly variations of pollutant concentrations throughout the year with medians, means, and lower and upper limits, including the first and third quartiles, are depicted. As stated out earlier (Russo et al. 2014), the results revealed that pollutants, except O_3_, reached their highest peak during cold season, especially in January and February; meanwhile, the lowest concentrations were recorded during warmer periods (May, June, and July). Furthermore, O_3_ evolution presents the highest values in January and April, and the lowest levels are found in November and December.

Analyzing the seasonal ratio aspects of CO, NO, and NO_2_ compared with PM_10_, higher values were obtained in winter and one of the possible reasons is that during winter the catalytic converters of vehicles take time to reach the optimal temperature, thereby resulting in increased CO emissions. On the other hand, the highest NO and NO_2_ levels were recorded in winter which can be attributed to increased fossil fuels for domestic heating and traffic since NO from traffic emissions is converted to NO_2_.

Moreover, in winter months, the pollutants which resulted from both natural and anthropogenic sources are trapped in the boundary layer due to frequent temperature inversions. During winter months, the atmospheric conditions are different, lower average wind speeds, lower temperatures, and lack of precipitations reduce surface vertical mixing which leads to limited dilution and dispersion.

The highest monthly means for PM_10_, O_3_, NO, NO_2_, NO_*x*_, SO_2_, and CO were 34.31, 67.31, 3.68, 20.92, 25.14, 5.83 (μg/m^3^), and 1.03 (mg/m^3^), respectively. Regarding the seasonality aspect, it can be seen that the highest mean values (increased pollution episodes) were reached for January and February as stated earlier. As depicted in Fig. 9, there is a clear tendency of concentration decreasing for major air pollutants, such as PM_10_, NO, NO_2_, NO_*x*_, SO_2_, and CO, especially during warm periods; hence, the lowest monthly mean values were observed in summer. This trend is in accordance with previous findings conducted worldwide, mainly high monthly means in cold and low monthly means in warm seasons (Russo et al. 2014). However, the monthly variation of O_3_ showed a significantly different pattern, in which the mean concentrations tend to be lower during the cold period, especially in November and December, than those in other months. One of the main reasons could be that due to reduced sunshine, low surface temperature and higher concentrations of primary pollutants (which scavenge O_3_) reduce the rate of photochemical formation of O_3_. The long-range transport, thermal inversion, atmospheric stability (boundary layer inversion), and heating during the cold period (biomass burning, peat fires) (Szép et al. 2019) likely play an important role; therefore, it is necessary to identify the potential source regions and long-range transport effect.

### Extreme episodes and long-term back-trajectories

**Fig. 10:**
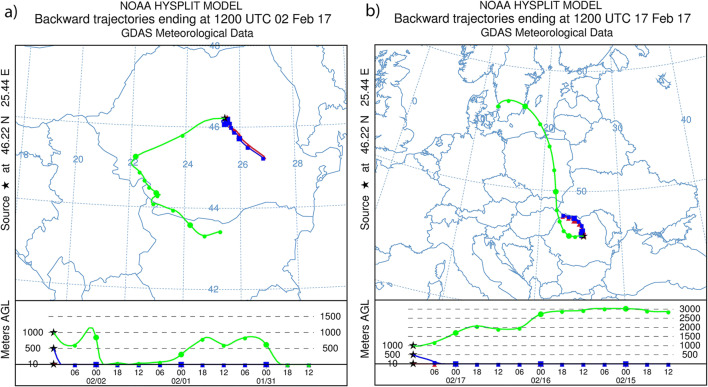
Backward trajectory analysis for 3 days on 02 (**a**) and 17 (**b**) February 2017 at the Ciuc basin

As already stated, one of the major factors of extreme pollution episodes is boundary layer inversion and this results in air stagnation, thus facilitating the occurrence of high levels of pollution, especially in the case of closed areas. Furthermore, the PM episodes are known to be caused by the regional and long-distance transport of pollutants as well (Xin et al. 2016; Li et al. 2017; Wang et al. 2017). Regarding the distribution of these episodes, a significantly greater frequency was reported in winter and spring; thanks to the stable, windless atmospheric conditions and long-range transport of pollution (Bessagnet et al. 2005; Vanos et al. 2015). In order to decipher the impact of long-range transport of pollutants on local PM_10_ levels and to gain a more clear insight into the possible origin of PM during extreme episodes, back-trajectory and cluster analyses were implemented. Based on these observations long-term simulations were carried out for the extreme episodes and the backward trajectories were calculated using the PC version of the HYSPLIT (Draxler and Rolph 2014). Our simulation results are depicted in Fig. 10 where we can observe the differences between the trajectories arriving at different heights. Figure 10 a shows the 3-day backward trajectories ending at Miercurea Ciuc (Ciuc basin) on 02 February 2017, which corresponds to the highest concentrations measured throughout the year. Figure 10 b shows the trajectories for the second period representing the second highest PM_10_ concentrations. To cover the air parcel movement, different altitudes were simulated: 10, 500, and 1000 m agl, respectively.

Briefly, the upper half shows the planar map, while the lower one shows the variation map of vertical heights of the trajectories above ground level.

According to the simulation results, it is obvious that air masses arriving at 1000 m traveled relative long distances, representing the fastest trajectories, while significantly shorter pathways were traveled by trajectories arriving at 10 and 500 m, respectively. Hence, PM_10_ concentration may be influenced by the intrusion of pollutants resulted from different areas. Even if the Ciuc basin is an intra-mountain depression, in some cases, the air masses may bring and load pollutants originating outside the urban area. Regarding the pathways obtained for periods with increased PM levels, the results indicate that the effect of the arrival heights in the case of 10 and 500 m agl on trajectories is not significant. Furthermore, on 02 February, the air masses had southwestern (1000 m) and southeastern (10 and 500 m) origins; meanwhile, on 17 February, the trajectories had mainly northwestern origins.

### Assessment of seasonal changes using cluster analysis approach

Given the reason that in general, a single trajectory has limitations in describing the path of an air parcel, a comprehensive analysis of the characteristics of trajectories is necessary since a single backward trajectory can identify only the movement of a single air parcel. Cluster analysis is a strong and essential technique known to be used to decipher the origin of air masses or the transport of pollutants arriving in a specific location. The HYSPLIT method is based on TSV (Lu et al. 2018), representing the total variance between clusters, and on the other hand on SPVAR, which is the minimum increase of spatial variance between cluster components (Kelly et al. 2012; Adame et al. 2014; Stein et al. 2015).

**Fig. 11:**
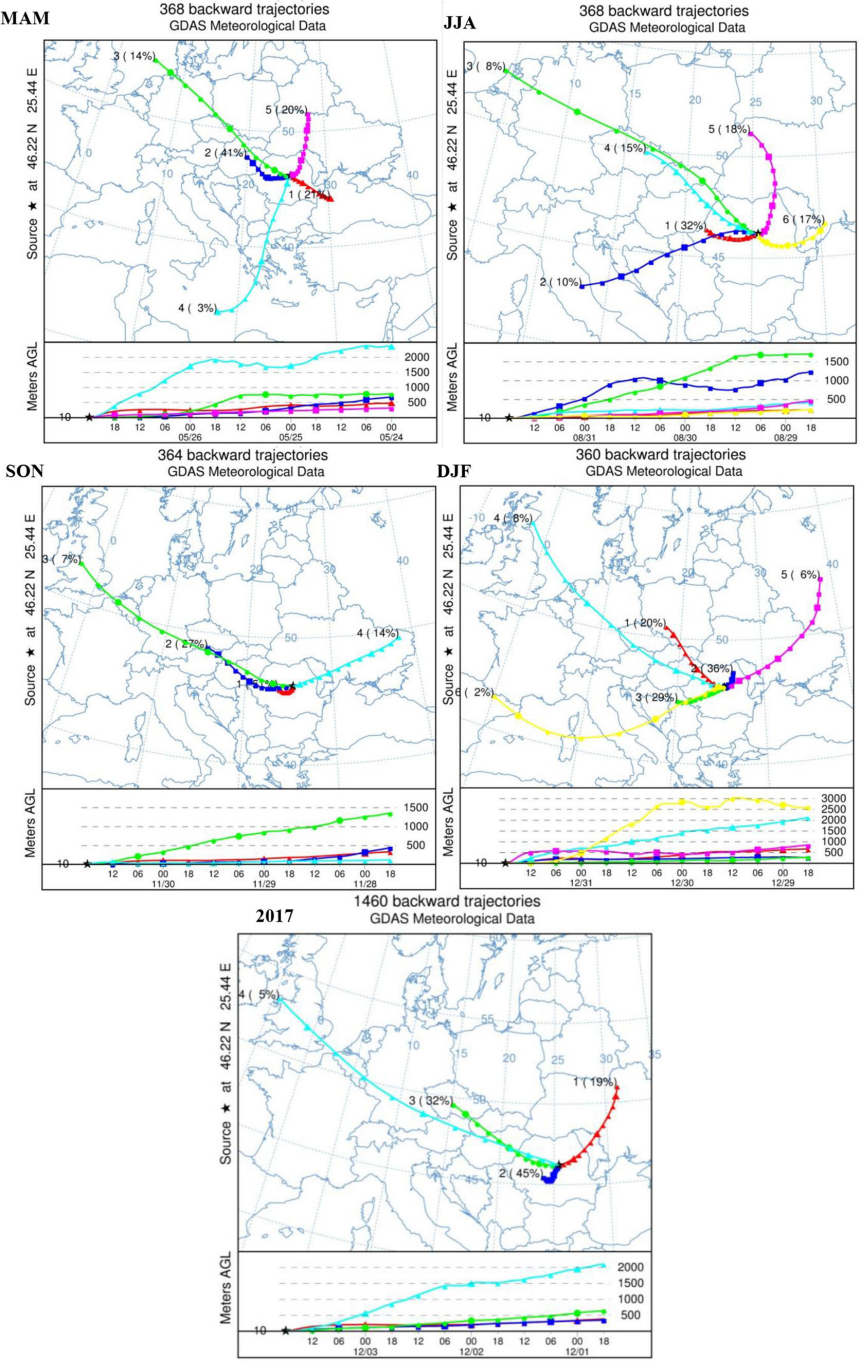
The individual centroids (cluster-mean trajectories) obtained for different seasons (spring, summer, autumn, and winter) and for the entire study period (2017)

The cluster-mean trajectories for each season, as well as the main clusters obtained for the entire study period, are shown in Fig. 11. Generally speaking, almost 50% of the clustered back trajectories have local and the other 50% continental origins. Regarding the transport speed of air masses, the longer cluster-mean trajectories represent faster air masses.

The optimal cluster number of each season was identified using TSV and those with the lowest value (Latif et al. 2012) were selected. The resulting number of clusters in each season was five, six, four, and six for spring, summer, autumn, and winter, respectively. Based on the HYSPLIT clustering outcomes, the trajectories arriving at the Ciuc basin at 10 m agl are found to be clustered in four major groups during the study period. Even if 72-h back trajectory simulation is sufficiently long to identify the main sources, additional analyses were also carried out for a much longer period; hence, 168 h was selected to cover the whole area of possible pollutions (not presented here). Broadly, the air masses that reached the region (during 2017), almost 50%, often come from the non-local areas and in many cases passing over heavily polluted regions. Analyzing the spatial distribution of trajectories, the results revealed that 45% (cluster 2) of the trajectories originated from the southwestern region from the study area with regional characteristics and slow-moving air masses over southeastern Transylvania. These trajectories are expected to be polluted because of the distance covered over land. Mean trajectories with long-range pathways, clusters 3 and 4 with a total occurrence frequency of 37%, have northwestern origins, and passed through the UK, Belgium, Germany, Austria, Hungary, Czech Republic, Slovakia, and Hungary, respectively, until they reached the study area.

Finally, 19% was attributed to cluster 1 with northeastern origin passing over countries like Ukraine and the Republic of Moldova.

As we expected, the fastest cluster frequency was low, representing only 5%. However, there are significant differences regarding the seasonal frequency distribution, which will be discussed in more detail below. The results revealed that trajectories having northwest directions have longer pathways and moved faster than those in other directions, while trajectories originating from southwest moved much slower. Taking the ourcomes into consideration, it can be stated that air parcels traveling over heavily industrialized regions may have been influenced by the atmospheric pollution in this region; however, further analyses are necessary to quantify the level of contribution. It should be emphasized that the most dominant cluster with almost 50% contribution of the cases has regional recirculation characteristics (centroid 2) and is characterized as the shortest path (short travel distance). Hence, the local and regional anthropogenic inputs are likely to be the highest and cluster 2 had the potential to be enriched by particulate matter. During simulations, 1460 backward trajectories were obtained which were grouped into four categories depending on the season. In order to see the differences between seasons, the clusters are analyzed separately.

#### Spring (March, April, May)

Clusters 3 and 4 are the longest (fastest) back trajectories, represent only 17%, and originated mainly over the North Sea at an altitude of over 700 m agl and over the Mediterranean Sea from an altitude of about 2000 m, respectively. Cluster 3 with a total occurrence frequency of 14% went through Germany, Czech Republic, Poland, Slovakia, and Hungary until they reached the study area in Romania. Fifty-two backward trajectories ending at various times were grouped into this centroid and the highest altitude reached by the air parcels during simulations was well above 3000 m agl. In the case of cluster 4, 3% frequency, 11 trajectories originated over the Mediterranean Sea from altitudes between 1000 and 5000 m agl and passed over Greece and Bulgaria (Fig. 11—MAM). The rest of the trajectories are grouped into three different clusters (1, 2, 5) with 21% frequency (78 backward trajectories, the air masses mainly originated from southeast—over the Black Sea), with 41% frequency (152 trajectories with north-western origins—cluster originated over Hungary), and with 20% frequency (75 trajectories with northern directions—cluster originated over Ukraine), respectively. On the other hand, these clusters have more regional characteristics and the trajectories mainly cover central and eastern Europe; therefore, it can be considered that these trajectories are important pathways that will contribute to particle concentrations.

#### Summer (June, July, August)

The optimal cluster number identified by the HYSPLIT was 6 and basically a similar tendency was observed between spring and summer; however, a few major differences were also identified. One of the most important observations is that clusters, during the summer season, have more westerly directions despite northerly directions observed in spring. In this case, the shortest pathway represents 32% (cluster 1); thus, the trajectories in this cluster originated from western Romania may indicate the regional pollution impact on air quality. The second group of clusters (5 and 6) represent the relatively short northeast and east trajectories, respectively, bringing air masses from Ukraine (35% frequency). By comparison, during the summer season on average trajectories started at lower altitudes compared with the spring season, the difference between highest altitudes (northwest airflow) was almost 1000 m.

#### Autumn (September, October, November)

During this period, the dominant air masses originated over the continent and approximately 86% (clusters 1, 2, 3) have western and northwestern origin. Furthermore, the main air masses arriving at the study area have westerly directions and 52% frequency was attributed to air masses with local origins. Trajectories associated with cluster 4 (14% frequency) originated mainly from the northeast and passed through southeast Ukraine and central Republic of Moldova.

#### Winter (December, January, February)

This is the coldest period with temperatures sometimes well below -25°C. Regarding the trajectories obtained for this period, continental air masses represent more than 90% of all trajectories: 20% northwester, 29% southwestern, and 42% northeastern, respectively. In winter, the most important air masses are included in clusters 2 and 3 accounting for about 65% of all trajectories, which may significantly influence the PM_10_ concentrations in the Ciuc basin due to the traveled distance and origin of the air masses (for the reasons presented earlier).

If clusters cover regions with negative water balance, the take-up and transport of particles are favored. According to our results, the air masses arriving at the study region come from the nearby or local regions ~ 50%, whereas half of the air masses have non-local origin. Considering the simulation outcomes, regional air masses are mainly distributed at approximately between 1500 and 2000 m agl over eastern Europe and the aerosol vertical distribution is mainly influenced by two factors: topography and trajectory distributions (Lu et al. 2018). Short trajectory patterns are expected to transport aerosols for different reasons: origins, re-suspension of road aerosol from street surfaces, and trajectory length. It is undeniable that the air parcel movement plays a key role on pollutant concentrations in the study area and to determine the major potential source regions, PSCF and CWT methods were implemented.

### Concentration-weighted trajectory

**Fig. 12:**
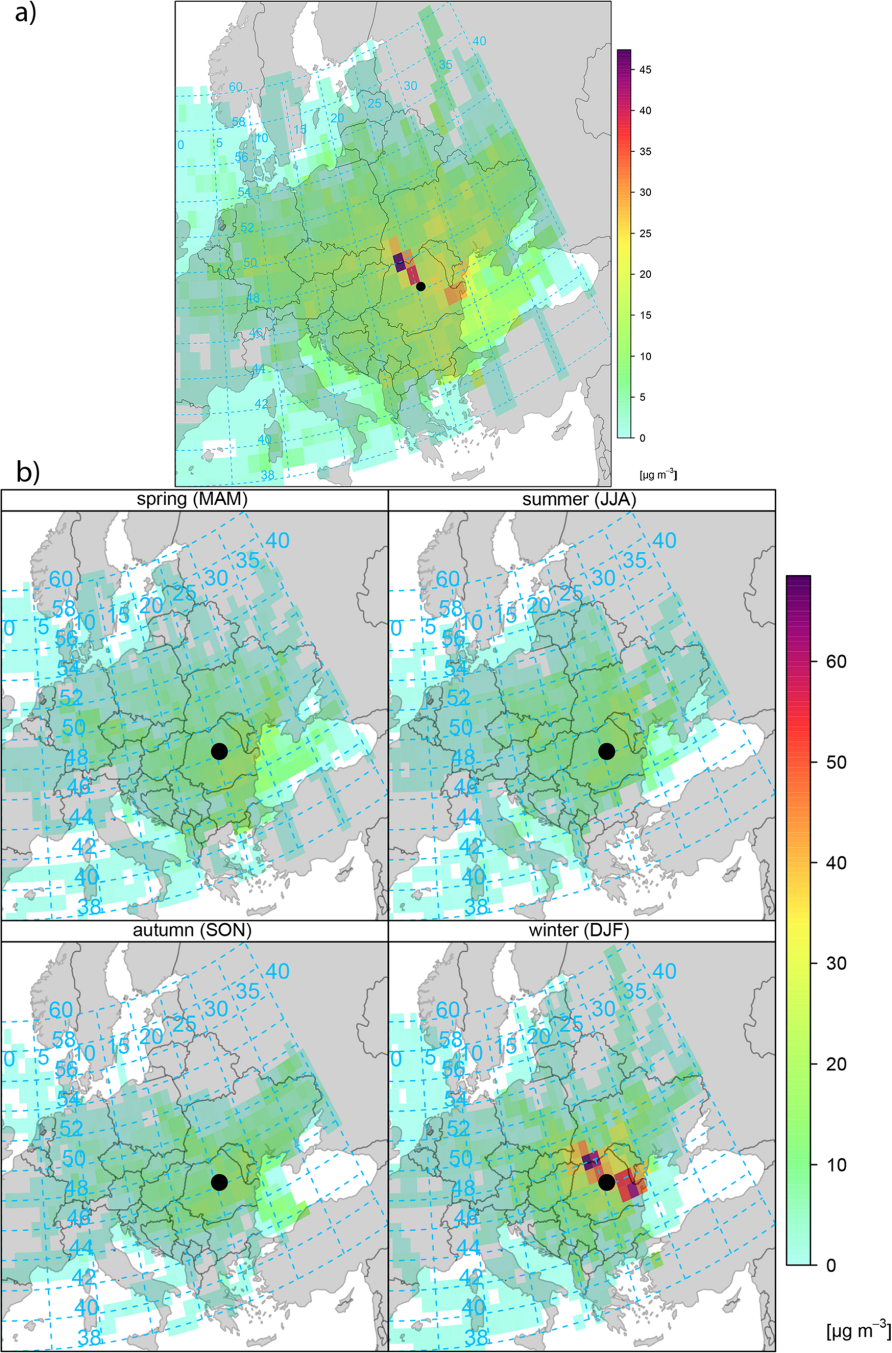
CWT plots for PM_10_ levels during 2017 (**a**) and for each season (seasonal variations) (**b**). Important potential source areas are dark-colored (≥ 90th percentile)

The CWT map for PM_10_ in the Ciuc basin during the study period and seasonal characteristics are shown in Fig. 12 and the output highlights major potential source areas affecting the regional PM_10_ concentrations.

The map outlines the main areas associated with relatively high PM_10_ concentrations recorded during 2017. The CWT result calculations revealed that in the case of PM_10_, the major potential source areas are found north, northwest, and southeast of the study region, respectively. Furthermore, the northeast and east areas such as the Republic of Moldova and Ukraine were identified as moderate source areas. Regarding the highest weighted concentrations, cells with CWT values above 45 μg/m^3^ were found northwest of the study site, including northwestern Romania and southwestern Ukraine. Hence, polluted air masses with potentially increased PM_10_ levels, originated from northwest, seem to have a negative influence on regional air quality. As stated out earlier (Dunea et al. 2016; Szép et al. 2017a), PM concentrations in Romania, especially in this region, were observed to be significantly higher in the cold period than in other seasons. On the other hand, the level of pollution is higher in urban areas and is significantly influenced by stable atmospheric conditions and reduced advection. Our CWT findings are in accordance with results reported in the literature, because strong seasonal variations were observed (Fig. 12b) due to the winter thermal inversion episodes (Szép et al. 2017b) indicating stable conditions with no or reduced removal of pollutants. During thermal stability and slow air movement, the pollutants originating especially from nearby regions may accumulate and may remain there for several days and this may explain the increased level of PM_10_ in winter. The weighted concentration of PM during the cold season was approximately 65 μg/m^3^. Meanwhile, in the three other seasons, the highest estimated CWT value was only ~ 15 μg/m^3^. Therefore, the plausible factors that could be attributed to increased PM_10_ levels are the presence of dominant regional winds (36%) and the increased emission rate of air pollutants as a result of enhanced biomass combustion from heating. Hence, the anthropogenic emission from biomass burning during the heating period seems to have a major impact on pollutant concentrations. These techniques can be used and make sense to analyze pollutant trajectories with regional component such as PM_10_; however, the analyses of gaseous species using CWT approach make little sense, since they are known to have local impacts.

**Fig. 13:**
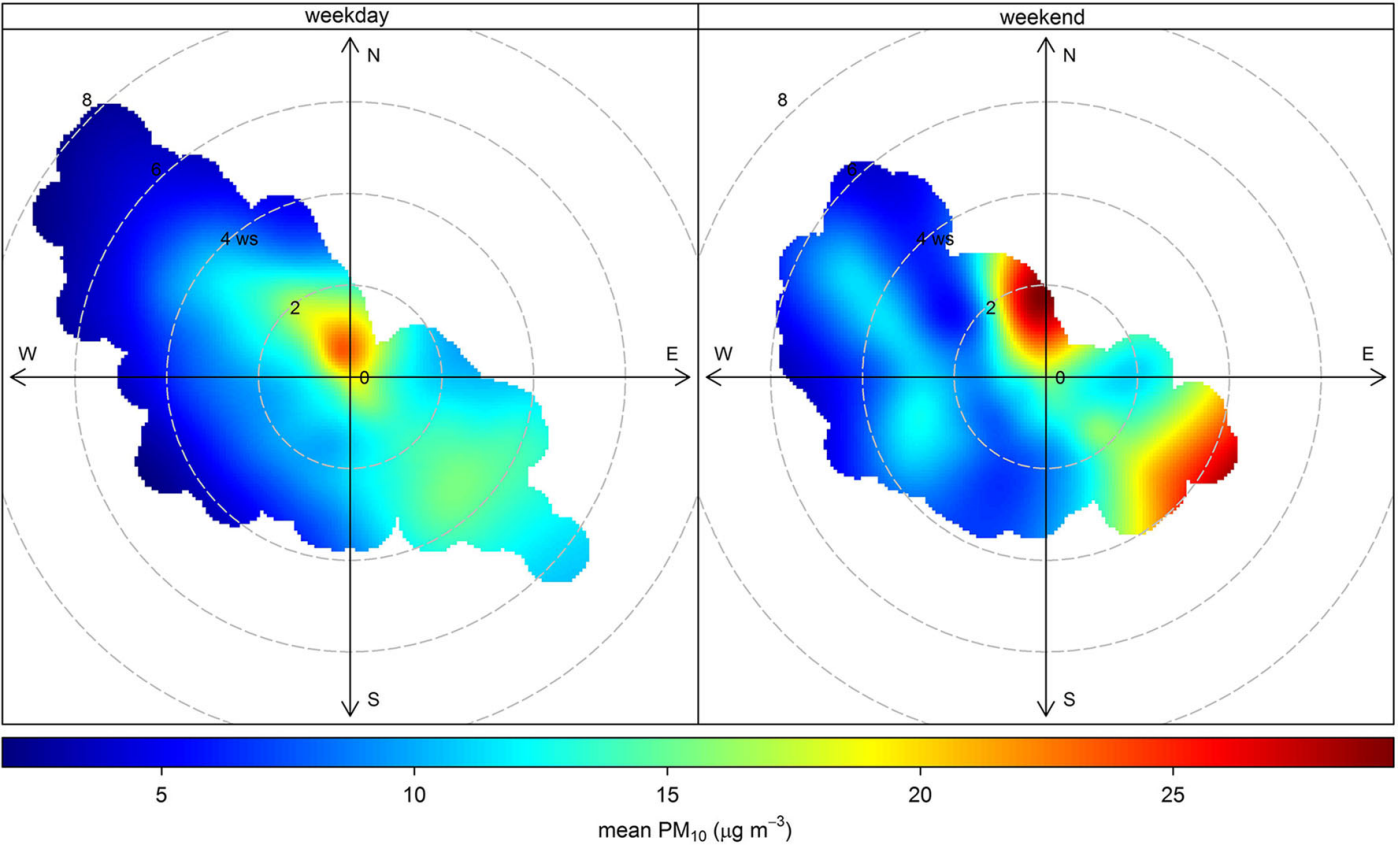
Bivariate polar plot of mean PM_10_ concentrations during weekdays/weekend

Furthermore, it is well known that there is a negative correlation between wind speed and wind direction (better ventilation and dilution during higher wind speed); however, it is important to identify the possible differences during the weekday/weekend PM. In order to decipher the weekend effect, the polarplot (Grange et al. 2016) function from openair package (Carslaw 2015) was used in the R programming environment. Plotting the data in polar coordinates may help us in the purposes of source identification since recent publications (Grange et al. 2016; Szulecka et al. 2017) have already proved the functionality of this method in determining potential sources of pollutants. Using bivariate polar plots, the concentration of different species is plotted with wind direction and wind speed in polar coordinate; hence, it is a highly efficient graphical technique to obtain directional information regarding potential emission sources. We analyzed the PM_10_ concentration variation differences between weekdays and weekends (Fig. 13) and we were interested if there are differences during the weekday/weekend PM_10_ concentrations. Regarding the weekday/weekend effect, highest values were recorded during weekends and interestingly the inflow was mainly from north and southeast, respectively, wind speed ranging from 2 to 3 m/s.

Generally speaking, the mean concentration for direction bins and wind speed confirms the main sources identified by CWT analyses and shows the multiple sources characteristics of PM_10_ in 2017. During weekday, the highest concentrations were recorded at low wind speed; meanwhile, weekend results show elevated concentrations with northwesterly winds at a wind speed of ~ 2 m/s and with southeasterly winds at a wind speed of ~ 4 m/s, respectively. Taken together, the results indicate a clear distinction in concentration levels as well as wind direction and speed between weekday and weekend.

## Conclusions

The present study has attempted to assess air pollution, the relationship between environmental parameters and pollutants in a closed basin as well as the possible source areas by measuring and analyzing the major air pollutant concentration during the year 2017. Furthermore, the seasonal variation of air pollution was deciphered including the extreme episodes as well. For example, monitored PM_10_ concentration levels were above the daily limit value (50 μg/m^3^) 11 times and the highest value was measured in February (132 μg/m^3^) during the coldest period. A clear seasonal pattern was identified for each pollutant, especially in the case of PM_10_, with highest concentrations in winter and lowest during the rest of the year. In general, the highest values were recorded during the coldest months, namely, January and February. One of the reasons why higher levels are measured during the cold season is that it is not only caused by heating (which has a seasonal characteristic) but also by the atmospheric stability and thermal inversions that characterize this region (intra-mountain basin). Therefore, with the heating time, the concentration of gaseous pollutants, SO_2_, NO_2_, NO, NO_*x*_, and on the other hand CO, increases due to thermal inversion (Szép et al. 2018, 2019). According to the results, the best air quality was recorded in summer months, while for winter, the ambient air quality is decreasing; nonetheless, the daily mean concentration of pollutants was below the legal limit. As stated out earlier (Chen et al. 2015a), the O_3_ concentration has a very different pattern (increased O_3_ values were recorded during summer) compared with other pollutants, which confirms different sources as well as the effect of solar radiation on O_3_ regeneration. A strong positive relation was identified between PM_10_ and other gaseous pollutants, while, as we expected, negative correlation was between temperature and wind speed. The 1-year average concentration (14.93 μg/m^3^) is decreasing compared with the last 5 years and it is far below the admissible limit (40 μg/m^3^). Moreover, the results suggest that the highest concentrations of all pollutants mainly occurred at low wind speed, except O_3_.

Furthermore, the potential effect of nearby emissions and long-range transport on PM_10_ concentration in the Ciuc basin was assessed. Cluster analysis can be used to decipher the main atmospheric circulation pathways, which may have a negative impact on the regional pollution levels. To understand the origins of air masses arriving at the study region, the dominant transport patterns (trajectories) and potential sources of PM_10_, 72-h long-range transport simulations were carried out using the well-known HYSPLIT model. The main centroids (clusters) over the receptor site and the potential pollution source areas, as well as the contribution to the PM_10_ loadings, were identified in the Ciuc basin. Most of the trajectories travel short distances (regional trajectories) ~ 45% of the cases. Possible source areas identified by CWT approach indicate northwest and southeast regions, namely southwestern Ukraine, the northwestern part of Romania, and southeast Romania (during winter), respectively. The simulation results revealed that during the extreme PM_10_ episodes (February), the air masses had manly southeastern and northwestern origins, respectively.

According to backward trajectories and CWT calculations, the aerosol concentration in the Ciuc basin can be affected by nearby sources due to the transport routes. Results indicate that even if the air masses mainly have southwestern origin, accounting 45% of all trajectories, the major pathways with high PM_10_ loadings have northwestern origins and short transport pathways. The dilution is higher during summer, thanks to the increased ventilation generated by strong winds from northwestern directions. Furthermore, the CWT findings revealed that air masses arriving from the southeastern and northwestern directions are the most polluted in the case of PM_10_; therefore, it might have an important influence on the Ciuc basin air quality. Regarding the seasonality aspects, it was observed that there was a seasonal variation of transport pathways and source areas; hence, the study region was mostly affected during winter by long-range transport and stable atmospheric conditions. The most frequent trajectories are the shortest and these trajectories that cover mid- and eastern Europe. One of the major differences between summer and other seasons is that the origin of trajectories is more westerly due to the active atmospheric dynamics. Moreover, the highest weighted concentrations, CWT values above 45 μg/m^3^, were found northwest of the study site (north, northwestern Romania) only during cold season; meanwhile, the PM_10_ concentrations were very low in spring, summer, and autumn, respectively. However, it should be emphasized that comparable high CWT values were attributed to trajectories with southeastern origins. Regarding the weekday and weekend effect, the bivariate polar plot results indicated a clear distinction in concentration levels and directions. Furthermore, a strong weekend effect in PM_10_ concentrations is noticeable, for example, higher concentrations were recorded with northwestern and southeastern winds.

It is obvious that the air quality may be altered not only by local anthropogenic activities but also by long-range transport of pollutants; hence, the improvement is a regional issue as well. The results demonstrate that local-scale meteorological conditions, long-range transport, and anthropogenic emissions (or nearby activities) play an important role in atmospheric pollution in this region. However, further investigations need to be carried out, e.g., multiple observations, in order to better understand the underlying processes that may take place under different atmospheric conditions, to improve the accuracy of air parcels movement, and to determine the possible impact on receptor site as well.

## Electronic supplementary material

ESM 1 (PDF 362 kb)

ESM 2 (PDF 397 kb)

ESM 3 (PDF 624 kb)
