# Supplementary material for: Major air pollutants seasonal variation analysis and long-range transport of PM10 in an urban environment with specific climate condition in Transylvania (Romania)
Source: Environ Sci Pollut Res Int. 2020 Jul 3;27(30):38181–99. doi: 10.1007/s11356-020-09838-2 (PMC7496053; doi:10.1007/s11356-020-09838-2)
Supplement: Supplementary file 1 — (PDF 362 kb) [file 11356_2020_9838_MOESM1_ESM.pdf]

Table 1. Values obtained for meteorological parameters and major air pollutants

	**WS**	**T**	**P**	**RH**	**CO**	**SO_2**	**NO**	**NO_2**	**NO_x**	**O_3**	**PM_{10}**
**Min**	0.00	-26.30	908.00	19.00	0.01	0.03	0.01	0.01	2.89	0.28	0.64
**Max**	7.40	34.76	950.90	100.00	3.86	16.11	35.21	67.48	108.66	163.33	132.58
**Average**	1.12	7.90	934.92	75.07	0.25	4.66	1.89	10.17	12.97	52.39	14.93
**Stdev**	0.93	10.26	5.97	17.57	0.55	0.99	2.32	7.74	9.71	25.99	15.94
**Median**	0.90	7.67	935.00	78.00	0.06	4.48	1.01	8.08	10.03	52.04	10.99

Abbreviations: WS – wind speed; T – temperature; P – atmospheric pressure; RH – relative humidity
