# Supplementary material for: Major air pollutants seasonal variation analysis and long-range transport of PM10 in an urban environment with specific climate condition in Transylvania (Romania)
Source: Environ Sci Pollut Res Int. 2020 Jul 3;27(30):38181–99. doi: 10.1007/s11356-020-09838-2 (PMC7496053; doi:10.1007/s11356-020-09838-2)
Supplement: Supplementary file 2 — (PDF 397 kb) [file 11356_2020_9838_MOESM2_ESM.pdf]

Table 2. Monthly Spearman correlation analysis of PM_{10} with the most important air pollutants

	Jan	Feb	Mar	Apr	May	Jun	Jul	Aug	Sep	Oct	Nov	Dec
CO	**0.436**	**0.605**	**0.645**	0.101	**0.608**	0.093	**0.442**	**0.475**	**0.441**	**0.595**	**0.518**	-0.188
SO_2	-0.238	0.313	0.292	**0.436**	0.140	0.092	0.249	**0.545**	**0.673**	**0.503**	0.029	-0.092
NO	**0.491**	**0.514**	0.088	**0.598**	**0.440**	0.249	0.338	-0.006	**0.467**	**0.562**	**0.624**	-0.040
NO_2	**0.468**	**0.605**	**0.507**	**0.725**	**0.674**	-0.312	**0.571**	**0.468**	**0.675**	**0.606**	**0.604**	-0.085
No_x	**0.477**	**0.627**	**0.672**	**0.750**	**0.678**	-0.281	**0.594**	**0.420**	**0.649**	**0.575**	**0.629**	-0.127
O_3	-0.010	-0.250	-0.159	-0.079	0.054	-0.167	0.150	**0.399**	0.270	**-0.413**	**-0.414**	0.341

Values in bold are different from 0 with a significance level alpha=0.05
