# Supplementary material for: Major air pollutants seasonal variation analysis and long-range transport of PM10 in an urban environment with specific climate condition in Transylvania (Romania)
Source: Environ Sci Pollut Res Int. 2020 Jul 3;27(30):38181–99. doi: 10.1007/s11356-020-09838-2 (PMC7496053; doi:10.1007/s11356-020-09838-2)
Supplement: Supplementary file 3 — (PDF 624 kb) [file 11356_2020_9838_MOESM3_ESM.pdf]

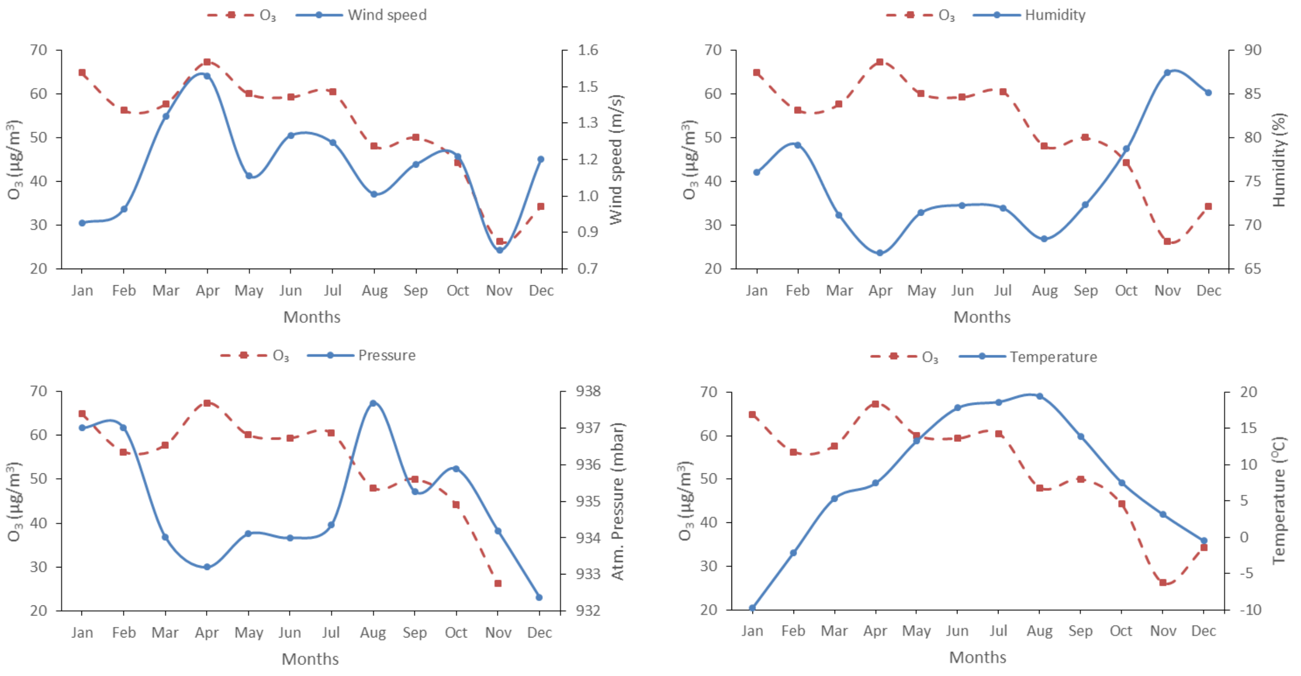

Fig.1 O_3, wind speed, humidity, atmospheric pressure and temperature monthly variations during the study period

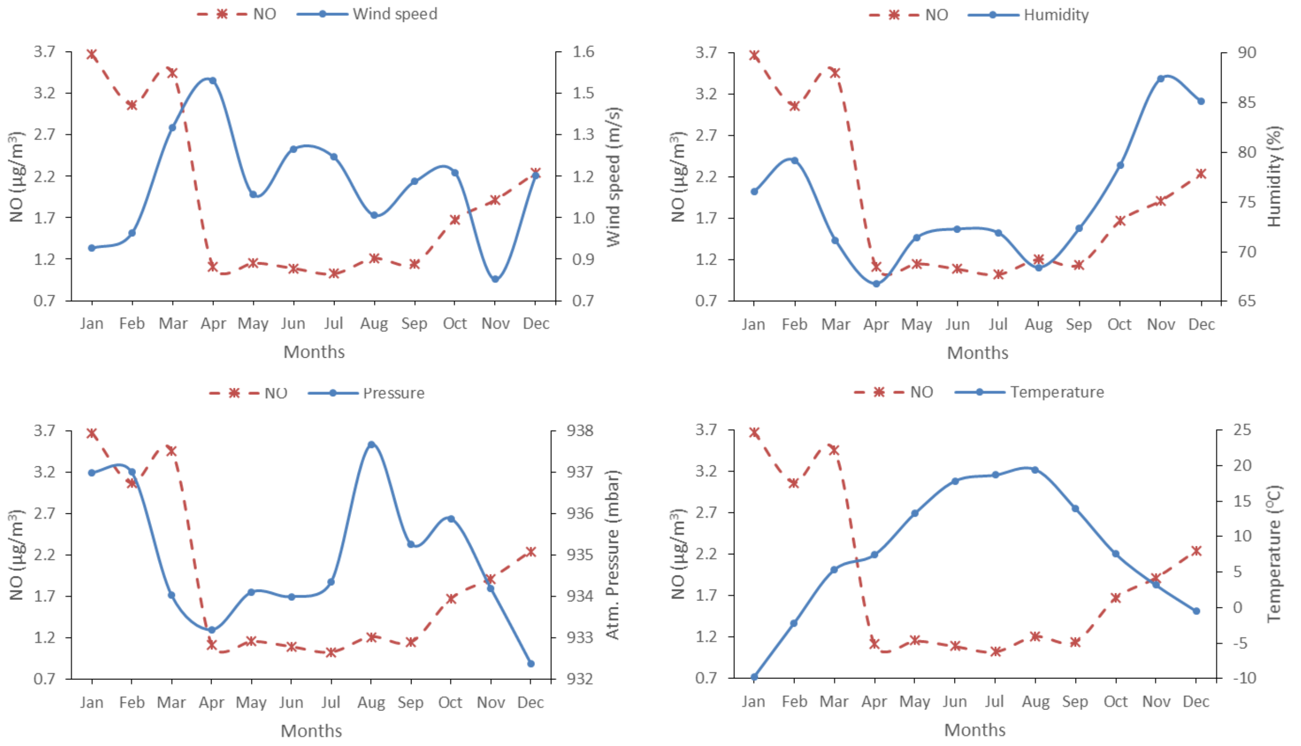

Fig.2 NO, wind speed, humidity, atmospheric pressure and temperature monthly variations during the study period

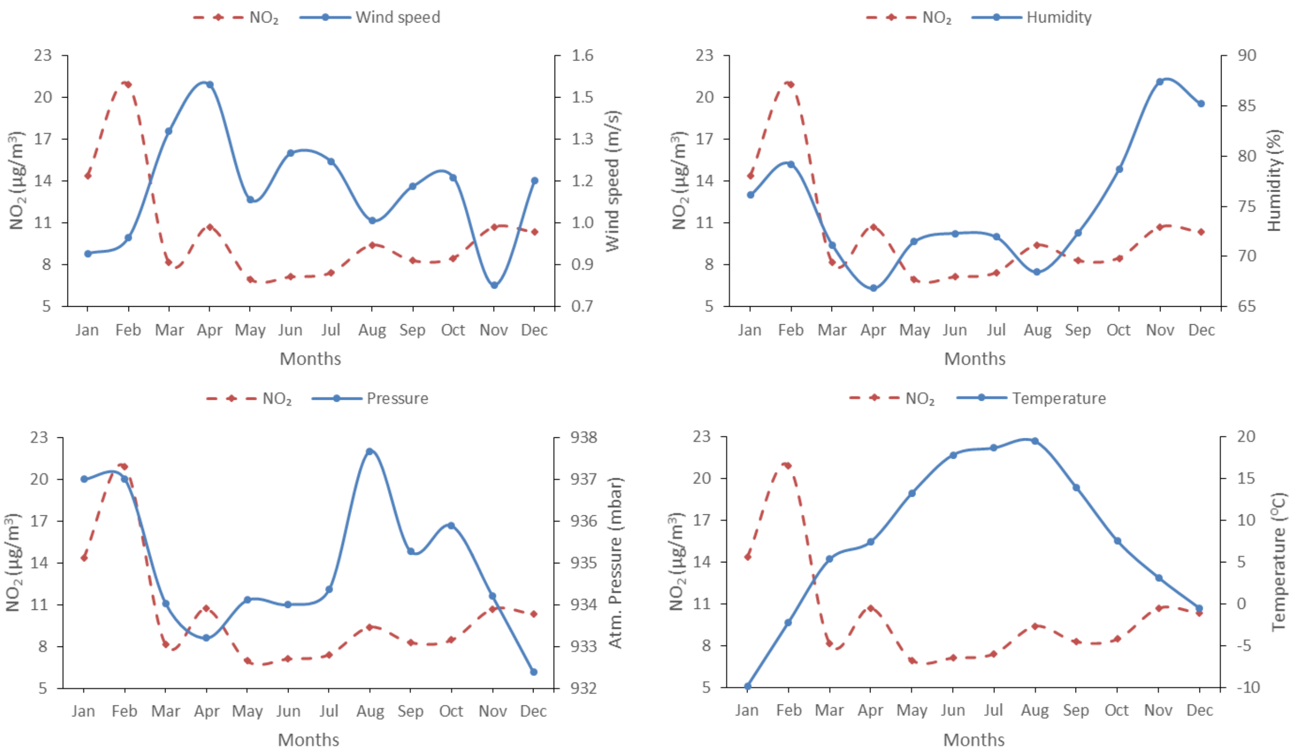

Fig.3 NO_2 and wind speed, humidity, atmospheric pressure and temperature monthly variations during the study period

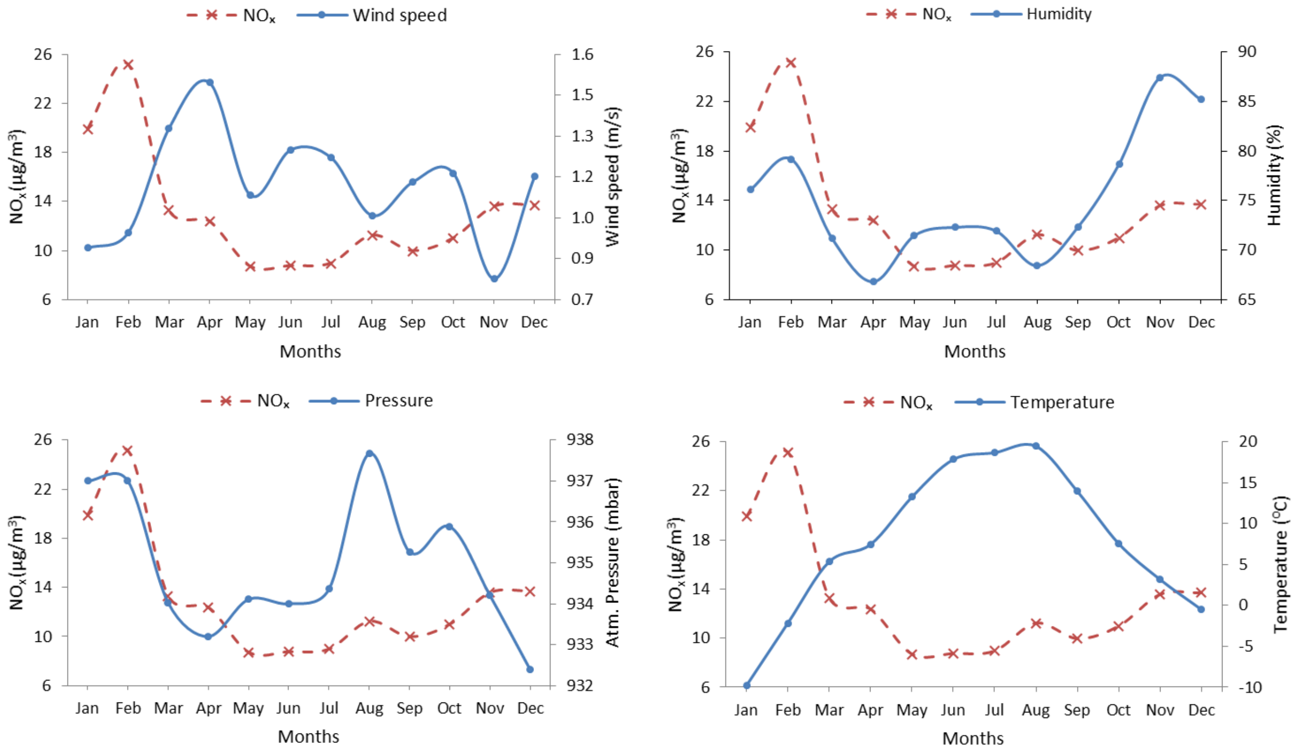

Fig.4 NOx and wind speed, humidity, atmospheric pressure and temperature monthly variations during the study period

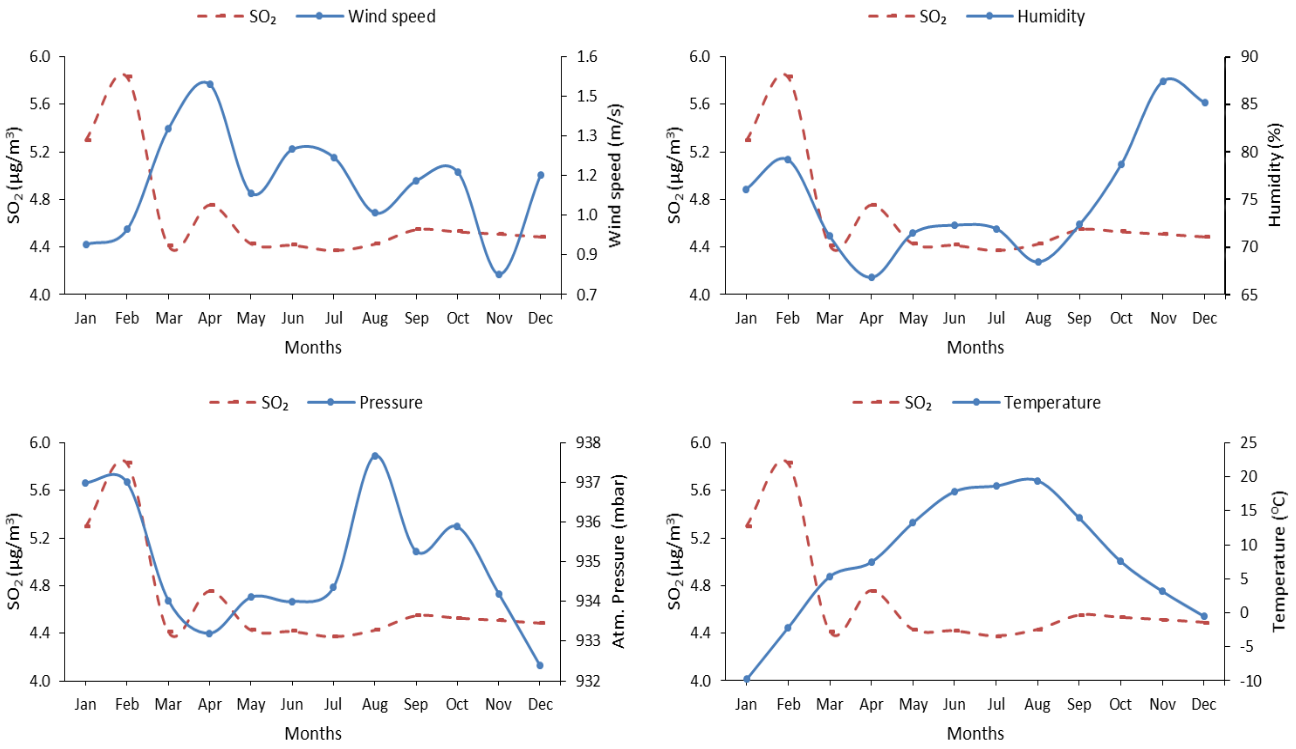

Fig.5 SO_2 and wind speed, humidity, atmospheric pressure and temperature monthly variations during the study period

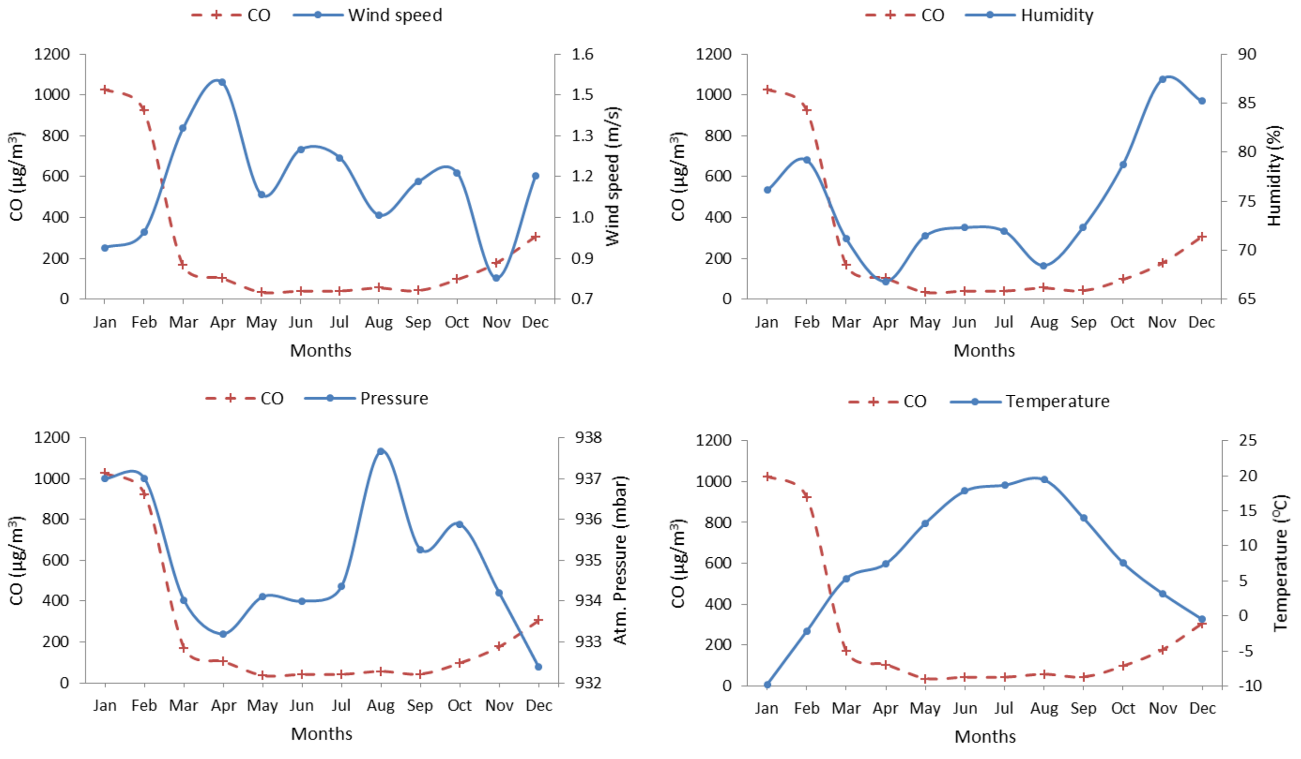

Fig.6 CO and wind speed, humidity, atmospheric pressure and temperature monthly variations during the study period
